# Prognostic Biomarkers in Pituitary Tumours: A Systematic Review

**DOI:** 10.17925/EE.2023.19.2.12

**Published:** 2023-08-09

**Authors:** Eirini Papadimitriou, Eleftherios Chatzellis, Anastasia Dimitriadi, Gregory A Kaltsas, Stamatios Theocharis, Krystallenia I Alexandraki

**Affiliations:** 1. First Department of Propaedeutic Medicine, Laiko Hospital, Medical School, National and Kapodistrian University of Athens, Athens, Greece; 2. Endocrinology Diabetes and Metabolism Department, 251 Hellenic Air Force and VA General Hospital, Athens, Greece; 3. Department of Pathology, Metropolitan Private Hospital, Athens, Greece; 4. First Department of Pathology, Medical School, National and Kapodistrian University of Athens, Athens, Greece; 5. Second Department of Surgery, Aretaieio Hospital Athens, Medical School, Athens, Greece

**Keywords:** Biomarkers, invasive adenomas, pituitary adenoma, pituitary tumours, prognostic biomarkers, recurrent adenomas

## Abstract

Pituitary tumours (PTs) are the second most common intracranial tumour. Although the majority show benign behaviour, they may exert aggressive behaviour and can be resistant to treatment. The aim of this review is to report the recently identified biomarkers that might have possible prognostic value. Studies evaluating potentially prognostic biomarkers or a therapeutic target in invasive/recurrent PTs compared with either non-invasive or non-recurrent PTs or normal pituitaries are included in this review. In the 28 included studies, more than 911 PTs were evaluated. A systematic search identified the expression of a number of biomarkers that may be positively correlated with disease recurrence or invasion in PT, grouped according to role: (1) insensitivity to anti-growth signals: minichromosome maintenance protein 7; (2) evasion of the immune system: cyclooxygenase 2, arginase 1, programmed cell death protein 1 (PD-1)/programmed death ligand 2, cluster of differentiation (CD) 80/CD86; (3) sustained angiogenesis: endothelial cell-specific molecule, fibroblast growth factor receptor, matrix metalloproteinase 9, pituitary tumour transforming gene; (4) self-sufficiency in growth signals: epidermal growth factor receptor; and (5) tissue invasion: matrix metalloproteinase 9, fascin protein. Biomarkers with a negative correlation with disease recurrence or invasion include: (1) insensitivity to anti-growth signals: transforming growth factor β1, Smad proteins; (2) sustained angiogenesis: tissue inhibitor of metalloproteinase 1; (3) tissue invasion: Wnt inhibitory factor 1; and (4) miscellaneous: co-expression of glial fibrillary acidic protein and cytokeratin, and oestrogen receptors α36 and α66. PD-1/programmed cell death ligand 1 showed no clear association with invasion or recurrence, while cyclin A, cytotoxic T lymphocyte-associated protein 4, S100 protein, ephrin receptor, galectin-3 , neural cell adhesion molecule, protein tyrosine phosphatase 4A3 and steroidogenic factor 1 had no association with invasion or recurrence of PT. With the aim to develop a more personalized approach to the treatment of PT, and because of the limited number of molecular targets currently studied in the context of recurrent PT and invasion, a better understanding of the most relevant of these biomarkers by well-d esigned interventional studies will lead to a better understanding of the molecular profile of PT. This should also meet the increased need of treatable molecular targets.

Pituitary tumours (PTs) are located in the *sella turcica*, which surrounds the adenohypophysis and neurohypophysis. PTs range from asymptomatic incidentalomas to symptomatic aggressive neoplasms, such as invasive neoplasms or pituitary carcinomas.^1^ Symptomatic lesions can be characterized either by hormonal overproduction or by dysregulation of hormone secretion, and in invasive lesions by tissue destruction and intracranial mass effects. PTs represent the second most common intracranial tumour, found in a rate of approximately 10–15% of intracranial tumours.^2^ Ninety per cent of PTs originate from adenohypophysial cells (pituitary adenomas [PAs]),^3^ and are found at autopsy in approximately 20% of the population, with an increasing prevalence in the USA over the past twenty years.^2,4^ An incidence of approximately 80 cases out of 100,000 residents of Banbury in Oxfordshire, UK has been reported, corresponding to a fourfold increase in prevalence compared with the previous decade.^5^ The incidence rate of PT in women is greater than men until the age of 50 years; after the age of 54 years, men have a greater incidence of PT.^6^ The majority of PTs show slow growth rates and remain within the *sella turcica* and/or displace the surrounding tissues. However, between 25% and 55% of PTs may show a more aggressive behaviour by infiltrating the surrounding structures, including the sphenoid and/or cavernous sinus, bones and, less frequently, nerves.^7–9^ They may also present an aggressive behaviour characterised by resistance to treatment, with patients experiencing early and frequent relapses.^10^

The previous 2004 World Health Organization (WHO) classification indicated that an increased number of mitoses, a Ki-67 labelling index of more than 3%, and extensive nuclear staining for p53 protein are indicators of aggressive behaviour.^11^ The most recent 2017 WHO classification takes into account tumour size and infiltration of the tumour in the cavernous and sphenoid sinuses, as assessed by magnetic resonance imaging, immunohistochemical type and markers of proliferation (increased mitotic activity and Ki-67).^12^ In addition, transcription factors such as pituitary-specific positive transcription factor 1 , steroidogenic factor 1 (SF-1) and TPit play a major role in determining tumour subtypes and have become part of the classification criteria.^13^

The fact that the biological behaviour of PTs cannot be predicted underlines the necessity to discover biomarkers that can predict the aggressive behaviour of PTs. The aim of this review is to report the novel biomarkers that are associated with tumour growth or invasion and might provide a possible prognostic value of tumour behaviour. Such prognostic value may assist clinicians in the determination of appropriate patient management and surveillance.

## Material and methods

### Protocol

#### Search strategy and selection of studies

This systematic review follows the Preferred Reporting Items for Systematic Reviews and Meta-Analyses (PRISMA) statement.^14^ The electronic databases of Medline (Pubmed) were reviewed systematically in August 2022, using appropriate controlled vocabulary and free search terms to identify studies evaluating prognostic biomarkers in PTs. The detailed search strategy vocabulary included the following: “pituitary neoplasms"[MeSH Terms] OR “pituitary neoplasms"[MeSH Terms] OR “pituitary lesions"[Other Term]) AND (“biomarkers"[MeSH Terms] OR “biomarkers, pharmacological"[MeSH Terms] OR “prognostic biomarkers"[Other Term] OR ((“immunohistologic"[All Fields] OR “immunohistological"[All Fields] OR “immunohistologically"[All Fields]) AND “markers"[Other Term]) OR “immunohistochemistry"[MeSH Terms] from 2016 to the present.

Titles, abstracts and full text (when appropriate) of all identified studies were screened for eligibility by three authors (EP, EC, AD). One author (EP) extracted from the studies the following pieces of information in a prespecified standardized Microsoft Excel spreadsheet: full reference; study identifiers; study design; eligibility; predefined outcomes; details on the outcomes of interest.

The search strategy was validated by KA. When EP raised a discrepancy, KA was consulted. All these steps were validated by a second reviewer (ST). Disagreement was resolved by discussion or adjudication by a third investigator (GK), if necessary.

#### Criteria for inclusion of studies in the systematic review

Studies fulfilling all of the following criteria were included in the systematic review:

I. studies published in English with prospective or retrospective design

II. studies that evaluated PTs of gonadotrophic, corticotrophic, somatotrophic or lactotrophic origins, mixed/plurihormonal adenomas, null-cell adenomas (NCAs) or non-functioning adenomas

III. studies that assessed biomarkers of a prognostic value by evaluating their presence or absence according to the aggressive behaviour of the PT (e.g. infiltration of surrounding tissues, recurrence)

IV. studies that compared the presence of biomarkers in PTs with healthy pituitary tissue, which have a potential diagnostic value or therapeutic targets for guiding personalized treatment

V. studies that evaluated gene mutations, proteomic profiling or DNA methylation were excluded from this review.

#### Study outcomes extracted for the systematic review

The primary outcome extracted from the selected studies was the presence or absence of immunohistochemichal biomarkers in PTs that show potential prognostic value. The secondary outcome extracted from the selected studies was any potential prognostic biomarker that may have gained importance in recent literature.

## Results

### Search strategy

**Figure 1:**
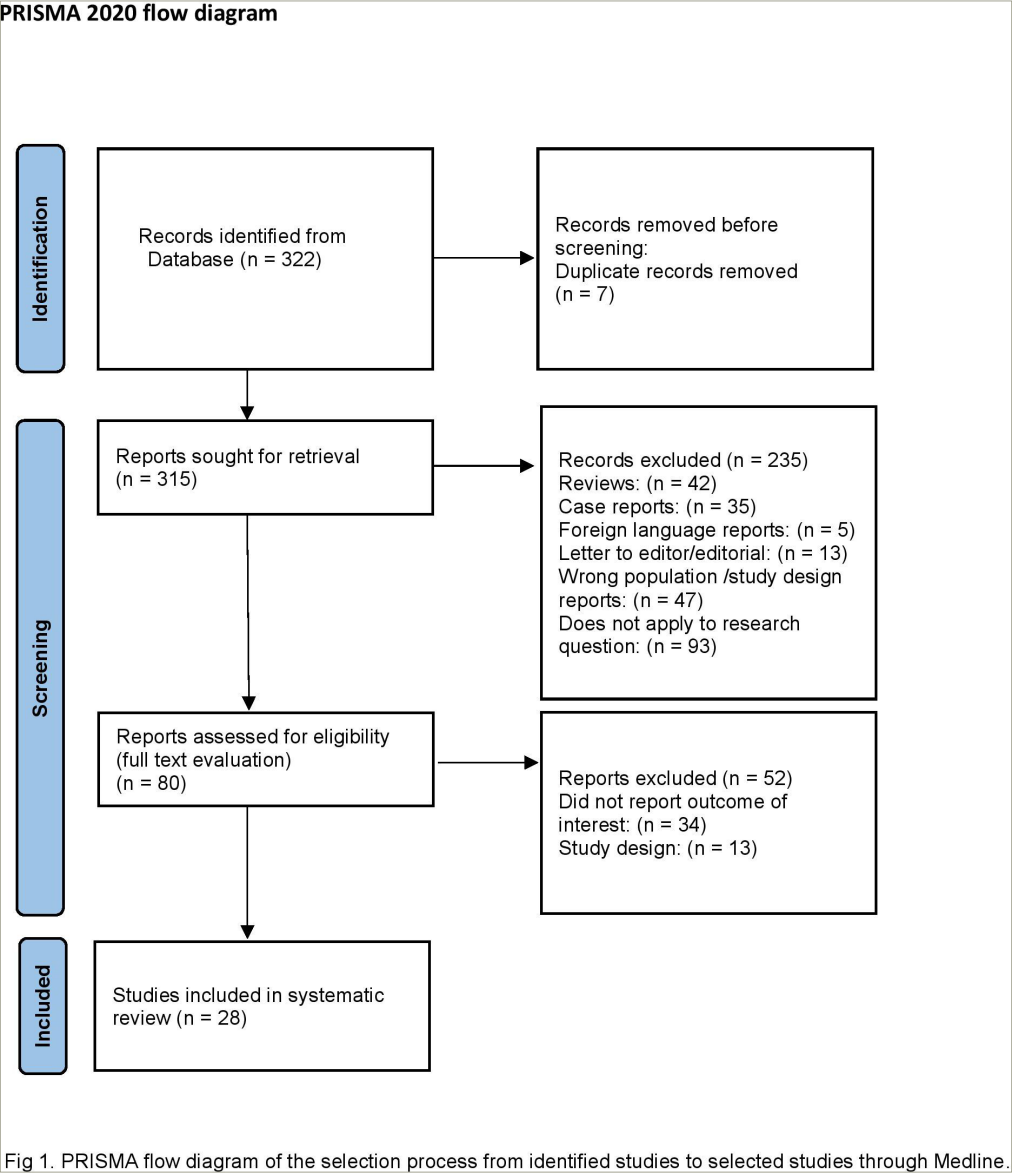
Preferred Reporting Items for Systematic Reviews and Meta-Analyses flow diagram of the selection process from identified studies to selected studies through Medline

The search strategy identified 322 references in Medline, which were screened by title and abstract. Of these references, 80 were deemed potentially eligible because they reported studies evaluating biomarkers in PT; 52 of the 80 studies were excluded because either the outcome was not precisely reported or their design did not fulfill the inclusion criteria. In the remaining 28 studies included in the present review, more than 911 PTs were evaluated. The selection process is described in *Figure 1*.

### Characteristics of the selected studies

Tissues were obtained from patients who underwent primary surgery for PA. Tumours that presented with pituitary apoplexy, necrosis or fibrosis or patients who took medications (such as cabergoline) were excluded from the studies. Study groups consisted of patients with invasive or recurrent PTs and were compared with patients with either non-invasive or non-recurrent PTs^15–29^ or normal tissues.^15,19,27,30^ Of the 28 studies included in this review, 20 described the study groups in detail. PTs were characterized as invasive or non-invasive according to Knosp classification, i.e. they are invasive when Knosp-score was ≥3 and/or the optic chiasm was compressed and non-invasive when Knosp score was 1–2.^15–17,22,24–26,28–36^ A recurrent tumour was defined as a new confirmed tumour that was diagnosed after complete resection, or the regrowth of a residual tumour after subtotal resection.^17,23,24,26–30,33,37^ Patients underwent follow-up with magnetic resonance imaging. In the prospective studies, the follow-up of the patients was between 2 and 11 years.

### Evaluated biomarkers

The biomarkers are reported in *Table 1*^15,17–35,37–45^ using the categories proposed by Hanahan and Weinberg.^46^ They suggest that most of the tumours have acquired the same sets of functional capabilities during their development, albeit there are various mechanistic strategies. According to their study, the hallmarks of cancer were originally six biological capabilities acquired during the multistep development of human tumours; this original hypothesis was established in 2000.^46^ In 2011, this was increased to eight biological capabilities and two enabling capabilities. The biological capabilities are:

### Minichromosome maintenance protein 7

Minichromosome maintenance (MCM) proteins are DNA-binding proteins expressed in the nucleus that are essential for the process of DNA replication. They are up-regulated in gliomas, meningiomas and prostate cancers.^47–51^ MCM-7 protein is found to be associated with cyclin D1-dependent kinase and may regulate the binding of this protein with the tumour suppressor retinoblastoma protein.^52^ MCM-7 expression has been found to predict poor clinical outcomes in various types of cancers.^53–56^

MCM-7 was evaluated in 70 non-functioning PAs (NFPAs), and it was found that MCM-7 expression was higher in the tumour progression group compared with the stable disease group over 6 years of follow-up (p<0.0001).^31^ The median MCM-7 expression was 7.4% (interquartile range [IQR] 2.4–15.2) in the group with tumour progression and 2.0% (IQR 0.6–5.3) in the stable disease group. The same study found a positive correlation of MCM-7 with Ki-67 and a negative correlation with age. These findings are consistent with a previous study that included 97 patients with both functioning PAs (FPAs) and NFPAs, where it was found that high MCM-7 expression was associated with an increased risk of tumour progression.^57^

### Cyclooxygenase 2 and arginase

Cyclooxygenase 2 (COX-2) is a key enzyme in the synthesis of prostaglandins, and when it is overexpressed, it can reduce the antitumour effect of the immune system by inhibiting the proliferation of B and T lymphocytes.^58^ Arginase 1 (ARG1) can process L-arginine in the local microenvironment and affect the function of T cells, resulting in immune escape.^59,60^

**Table 1:** Evaluated biomarkers according to their biological capability, association with tumour behaviour, outcome of the studies and method used*

Biomarkers	Association with invasion/ recurrence	Studies	Evaluated tissues and outcomes	Method used
Insensitivity to anti-growth signals				
MCM-7 protein	(+)ve	Hallen et al., 2021^31^	70 NFPAs; association with tumour progression and Ki-67	IHC
TGF-β1	(-)ve	Li et al., 2021^24^	Somatotroph PΤ (13 invasive; 32 non-invasive): ↓TGF-β1 protein level in invasive versus non-invasive (p<0.01)	Western blot
		Gu et al., 2018^25^	NFPAs (19 invasive; 23 non-invasive): ↓ TGF-β expression in invasive versus non-invasive NFPAs (p<0.05)	IHC; qRT-PCR; Western blot
		Zhu et al., 2018^26^	NFPAs (59 recurrent; 45 non-recurrent): ↓ TGF-β1 expression in recurrent versus non-recurrent (p<0.001)	qRT-PCR; Western blot
Smad proteins	(-)ve	Li et al., 2021^24^	Somatotroph PΤ (13 invasive; 32 non-invasive) lower phospho-Smad3 protein level in invasive versus non-invasive (p<0.01)	IHC; Western blot
Cyclin A	No association	Lamback, 2020^29^	31 NFPAs: no significant difference between invasive and non-invasive	qRT-PCR
Evasion of the immune system				
COX-2	(+)ve	Zhao et al., 2020^30^	55 PTs (30 NFPAs, 25 FPAs) and 10 normal pituitaries: ↑ expression in PTs versus normal pituitaries (p<0.001); no significant difference in NFPA versus FPAs	IHC; Western blot
		Akbari et al., 2020^15^	71 PTs (33 invasive; 38 non-invasive); 20 normal pituitaries; ↑ expression in invasive versus non-invasive PTs (p=0.04); ↑ expression in PTs versus normal pituitaries (p=0.0001); ↑ expression in NFPAs & FPAs versus normal pituitaries, but not statistically significant	qRT-PCR
ARG1	(+)ve	Zhao et al., 2020^30^	55 PTs (30 NFPAs, 25 FPAs) and 10 normal pituitaries; ↑ expression in PTs versus normal pituitaries (p<0.001); ↑ expression in NFPAs versus FPAs (p=0.005)	IHC; Western blot
PD-1/PD-L2	(+)ve	Xi et al., 2021^27^	60 PTs (43 recurrent/invasive; 17 non-recurrent/non-invasive); ↑ levels in recurrent/invasive PTs (p<0.0001) versus normal pituitaries	qRT-PCR
CD80/CD86	(+)ve	Xi et al., 2021^27^	60 PTs (43 recurrent/invasive; 17 non-recurrent/ non-invasive); ↑ levels in recurrent/invasive PTs CD80 (p=0.0035); CD86 (p=0.004) versus normal pituitaries; ↑ CD86 levels (p=0.0035) in recurrent/invasive versus non-recurrent/ non-invasive	qRT-PCR
PD-1/PD-L1^38^	No clear association	Xi et al., 2021^27^	60 PTs (43 recurrent/invasive; 17 non-recurrent/non-invasive); no significant difference in recurrent/invasive versus normal pituitaries	qRT-PCR
		Uraki et al., 2020^34^	37 non-invasive; 36 invasive NFPAs: significantly ↓ expression of PD1/PD-L1 (p<0.05) in invasive versus non-invasive	qRT-PCR
		Zhao et al., 2020^30^	55 PTs (NFPA, FPA); 10 normal pituitaries: ↑ expression of PD1/PD-L1 in NFPAs & FPAs (p<0.001) versus normal pituitaries	IHC; Western blot
		Mei et al., 2021^39^	72 PTs: ↑ expression of PD1/PD-L1 in FPAs (p<0.01) versus NFPAs	IHC
		Zhou et al., 2020^35^	115 PTs: PD-1 (p<0.001) & PD-L1 (p<0.01) ↑ expression of PD1/PD-L1 in FPAs versus NFPAs (especially somatotroph)	IHC
		Suteau et al., 2020^40^	139 PTs: no significant difference between FPAs and NFPAs (p=0.26)	IHC
CTLA-4 immunotherapy target (ipilimumab)	No association	Xi et al., 2021^27^	60 PTs (43 recurrent/invasive; 17 non-recurrent/non-invasive); no significant difference in recurrent/invasive versus normal pituitaries	qRT-PCR
		Zhou et al., 2020^35^	115 PTs: no significant difference between FPAs and NFPAs (p>0.05)	IHC
S100 protein	No association	Ilie et al., 2022^32^	54 PTs; four normal pituitaries: no association with invasion; ↓ S100 expression associated with Ki-67 index ≥3, mitosis count >2/10/HPF	IHC
Sustained angiogenesis				
ESM1	(+)ve	Wang S et al., 2019^22^	94 NCAs (45 invasive; 49 non-invasive): (+)ve association ESM1 and tumour invasion (p=0.002).	IHC
FGFR	(+)ve	Durcan et al., 2022^18^	161 PTs: ↑ FGFR-4 expression in PTs without remission and residual lesions (p<0.05)	IHC
MMP-9	(+)ve	Liu et al., 2018^19^	55 corticotroph PTs: ↑ MMP-9 expression in recurrent PTs versus non-recurrent (p<0.05); no expression in two normal pituitaries	IHC
		Guo et al., 2019^21^	108 PTs (58 invasive; 50 non-invasive): ↑ MMP-9 expression in invasive versus non-invasive (p<0.05)	IHC, qRT-PCR; Western blot
PTTG	(+)ve	Wang H et al., 2019^22^	30 NFPAs (15 invasive; 15 non-invasive): ↑ mRNA expression of PTTG in invasive versus non-invasive (p<0.01)	qRT-PCR; Western blot
		Gruppetta et al., 2017^37^	74 PTs: (+)ve association PTTG expression and recurrence NFPA and FPA	IHC; qRT-PCR; Western blot
		Trott et al., 2019^23^	56 NFPAs: ↑ PTTG expression invasive versus non-invasive (p=0.022)	IHC
		Liu et al., 2018^19^	55 corticotroph PTs: no significant difference of PTTG expression between recurrent and non-recurrent PTs	IHC
TIMP-1	(-)ve	Guo et al., 2019^21^	108 PTs (58 invasive; 50 non-invasive): ↑ expression of TIMP-1 non-invasive versus invasive (p<0.05)	IHC; qRT-PCR; Western blot
Self-sufficiency in growth signals				
EGFR	(+)ve	Rai et al., 2021^17^	102 NFPAs: ↑ expression phosphorylated EGFR recurrent versus non-recurrent (p<0.001)	IHC
EPH	No association	Papadimitriou et al., 2022^41^	18 PTs: ↑ expression mainly EPH-A4 & EPH-A5, -B2, -B4	IHC
Galectin-3	No association	Bima et al., 2021^28^	24 non-invasive; 12 invasive prolactinomas: no significant difference invasive versus non-invasive	IHC
S100 protein^32^	No association	As mentioned above		
NCAM	No association	Marques et al., 2021^42^	24 PTs; five normal pituitaries: no association with invasion	IHC
PTP4A3	No association	Moyano Crespo et al., 2021^43^	34 PTs: significant association with size (p=0.042)	IHC
Tissue invasion				
MMP-9^19,21^	(+)ve	As mentioned above		
TIMP-1^21^	(-)ve	As mentioned above		
Fascin protein	(+)ve	You et al., 2021^20^	30 PTs: ↑ expression fascin protein invasive versus non-invasive	IHC
WIF1	(-)ve	Zhu et al., 2018^26^	104 NFPAs (59 recurrent; 45 non-recurrent): ↓ expression WIF1 recurrent versus non-recurrent (p<0.001)	qRT-PCR; Western blot
Miscellaneous				
SF-1	No association	Hickman et al., 2021^33^	31 gonadotrophic PTs (11 non-recurrent; 20 recurrent). Patchy SF-1 staining associated with recurrence versus diffuse staining (p=0.0007)	IHC
Co-expression GFAP and cytokeratin	(-)ve	Wiesnagritski et al., 2021^44^	326 PTs; 13 normal pituitaries: co-expression GFAP and cytokeratin associated ↓ recurrence rate (7.7%) versus adenomas without co-expression (17.8%)	IHC
ERα36 and ERα66	(-)ve	Mahboobifard et al., 2020^45^	62 prolactinomas: ↓ ERα36 & ↓ ERα66 expression associated with tumour invasion	IHC

*The main role of each biomarker is discussed in detail in the text.

*ARG1 = arginase 1; CD = cluster of differentiation; COX-2 = cyclooxygenase 2; CTLA-4 = cytotoxic T-lymphocyte-associated protein 4; EGFR = epidermal growth factor receptor; EPH = ephrin receptor; ER = oestrogen receptor; ESM1 = endothelial cell-specific molecule 1; FGFR = fibroblast growth factor receptor; FPA = functioning pituitary adenoma; GFAP = glial fibrillary acidic protein; HPF = high power field; IHC = immunohistochemistry; MCM-7 = minichromosome maintenance protein 7; MMP-9 = matrix metalloproteinase-9;*

*NCAM = neural cell adhesion molecule; NFPA = non-functioning pituitary adenoma; PD-1 = programmed cell death protein 1; PD-L = programmed cell death ligand; PT = pituitary tumour; PTP4A3 = protein tyrosine phosphatase 4A3; PTTG = pituitary tumour transforming gene; qRT-PCR = quantitative reverse transriptase polymerase chain reaction; RB = retinoblastoma protein; SF-1 = steroidogenic factor 1; TGF-β1 = transforming growth factor β1; TIMP-1 = tissue inhibitor of metalloproteinase 1; (+)ve = positive; (-)ve = negative; VEGF = vascular endothelial growth factor; WIF1 = Wnt inhibitory factor 1.*

COX-2 and ARG1 were evaluated in 25 FPAs, 30 NFPAs and 10 healthy pituitary tissues. There was a significant difference in the expression of COX-2 and ARG1 in PTs compared with healthy tissues (p<0.001).^30^ There was no significant difference in the expression of COX-2 between FPAs and NFPAs, whereas expression of ARG1 was higher in NFPAs compared with FPAs (p=0.005). Another study investigated the expression of COX-2 in 71 PAs (33 invasive PAs and 38 non-invasive PAs; 30 NFPAs and 41 FPAs) and in 20 healthy pituitary tissues.^15^ The study found that higher levels of COX-2 were expressed in PTs compared with normal pituitary tissue (p=0.0001). Moreover, the expression of COX-2 was significantly increased in invasive tumours compared with the non-invasive tumours (p=0.04).^15^ The same study evaluated prostaglandin E2, which has a positive role in cell proliferation, angiogenesis and inflammation, and a higher expression was observed in PTs, NFPAs and invasive PTs compared with normal pituitaries, FPAs and non-invasive PAs, respectively. In contrast to the previous study,^30^ the expression of COX-2 was significantly elevated in NFPAs compared with FPAs (p=0.001).^15^

### Endothelial cell-specific molecule 1

Endothelial cell-specific molecule 1 (ESM1) is a dermatan sulfate proteoglycan secreted by endothelial cells that are regulated by possible markers of angiogenesis, such as vascular endothelial growth factor (VEGF) and fibroblast growth factor (FGF).^61–63^ ESM1 is a biomarker associated with tumour progression in various types of tumours, including lung, liver, brain, kidney and gastric tumours. Its overexpression has been associated with poor prognosis,^64–67^ and there is indirect evidence of it being a poor prognostic biomarker in PT.^68^ ESM1 was investigated in 94 NCAss (45 invasive NCAs and 49 non-invasive NCAs), and a positive association was observed between ESM1 expression in vascular endothelial tissues and tumour invasion (p=0.002).^22^ Low expression of ESM1 was observed in 19 invasive NCAs (42.2%) and in 26 non-invasive NCAs (57.8%), whereas high expression was observed in 30 invasive NCAs (61.2%) and in 19 non-invasive NCAs (38.8%) (p=0.065).^16^

### Tyrosine kinase receptors

#### Epidermal growth factor receptor protein

Epidermal growth factor receptor (EGFR) is a transmembrane glycoprotein belonging to the human epidermal growth factor receptor (HER) family of tyrosine kinase receptors (TKRs), which promote multiple signalling cascades for cellular survival.^69^ Both neoplastic and normal pituitary tissues express EGFR and phosphorylated EGFR (pEGFR). Overall, NFPAs have higher EGFR and pEGFR expression than FPAs, thus pEGFR expression is more amplified in neoplastic pituitary tissue compared with normal pituitary tissue.^70^ The expression of pEGFR was investigated in recurrent NFPAs (n=47) and in non-recurrent NFPAs (n=55), and a highly significant difference was reported (p<0.0001). pEGFR positivity was greater in a higher number of recurrent NFPA and the H-scores were also higher in recurrent NFPA compared with non-recurrent (117 ± 6.0 versus 71.2 ± 3.7; p<0.0001).^17^ The H-score is determined by adding the results of multiplication of the percentage of cells with staining intensity ordinal value (scored from 0 for 'no signal' to 3 for 'strong signal') with 300 possible values. The study also reported that high expression of pEGFR predicted higher probability of recurrence in NFPA (i.e. hazard ratio [HR] 4.9, confidence interval 2.8–8.8; p<0.0001).

#### Fibroblast growth factor receptor 4

Fibroblast growth factor receptor 4 (FGFR4) belongs to the TKRs that regulate cellular pathways involved in proliferation, differentiation and survival.^71^ A prospective study investigated the expression of FGFR4 in 161 PAs, with the patients followed-up for a period of 61 months. The median H-scores for FGFR4 were higher in patients without remission and those with residual lesion compared with those with remission (p<0.05).^18^ Moreover, PTs with Ki-67 expression ≥3% had higher FGFR4 expression levels than those with <3% expression (p=0.002), and a weak positive correlation between H-score and Ki-67 (p=0.011; *r*=0.201) was reported.^18^

#### Ephrin receptors

**Figure 2:**
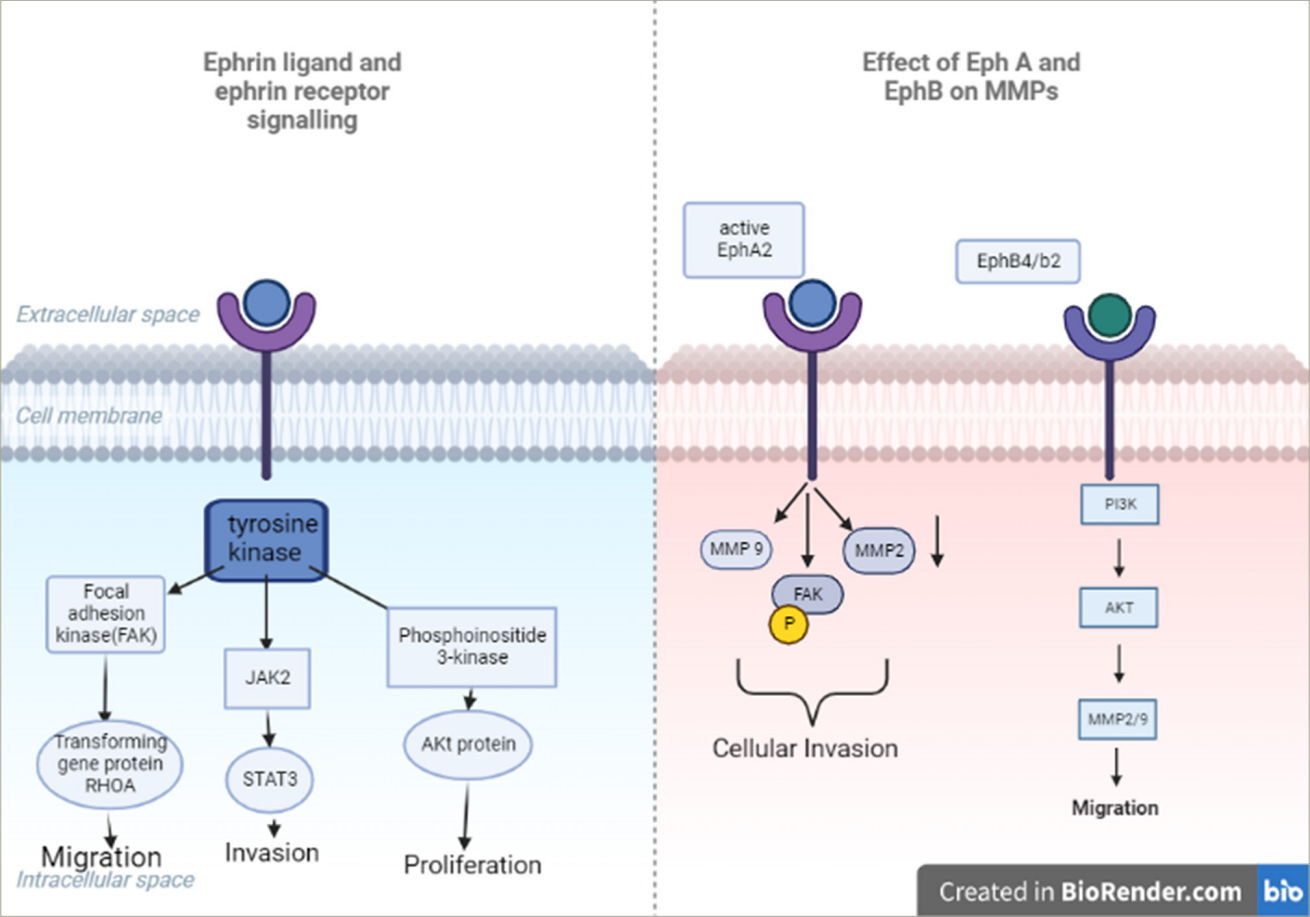
Ephrin receptor signalling and its effect on matrix metalloproteinase

Ephrin receptors (EPHs) comprise the largest known subfamily of TKRs. They bind to and interact with EPH-interacting proteins. They have a role in tumour growth, invasion, angiogenesis and metastasis of several neoplasms. Preliminary data indicate a high prevalence of EPH-A4 and a lower expression of EPH-A2 in neuroendocrine neoplasms.^72^ The expression of EPH-A4, -A5, -B2 and -B5 were evaluated in 18 PTs (seven somatotropic and two corticotropic adenomas, eight nonfunctioning macro-adenomas and one resistant prolactinoma) by immunohistochemistry.^41^ The data reported, for the first time, the increased expression of mainly EPH-A4 and, to a lesser extent, EPH-A5, -B2 and -B4 in pituitary lesions. A cytoplasmic (17/18) and nuclear (13/18) pattern of immunostaining for EPH-A 4 and a cytoplasmic pattern for EPH-A5, -B2 and -B4 was noted. All corticotropic and somatotropic adenomas were positive for EPH-A4 for both patterns, whereas positivity for EPH-A5 (4/18) and EPH-B2 (1/18) was noted in NFPAs with cytoplasmic pattern. The H-score for EPH-A4 expression ranged from 30–255, whereas for EPH-A5, -B2 and -B4 the range was lower (10–65) (*Figure 2*).^41^

### Matrix metalloproteinase-9 and tissue inhibitor of metalloproteinase 1

Matrix metalloproteinases (MMPs) are capable of degrading all kinds of extracellular matrix proteins that may play an important role in cell proliferation, migration, angiogenesis and apoptosis. MMP-9 may play an important role in angiogenesis and neovascularization and appears to be involved in the remodelling associated with malignant glioma neovascularization.^73^ MMP-9 and tissue inhibitor of metalloproteinase 1 (TIMP-1) were investigated in 58 invasive PTs and in 50 non-invasive PTs, and it was found that positive expression of MMP-9 in invasive PAs was significantly higher than in non-invasive PTs, whereas positive expression of TIMP-1 was relatively high in non-invasive PTs, with the differences being statistically significant (p<0.05).^21^ In another study, MMP-9 was evaluated in 27 recurrent adrenocorticotropic hormone (ACTH)-secreting PAs and in 28 non-recurrent ACTH-secreting PTs and in two normal tissues. Recurrent ACTH-secreting PTs were found to express greater levels of MMP-9 and showed a shorter recurrence-free interval.^19^ No expression of MMP-9 was detected in the two normal pituitary tissues.^19^ A previous study also reported that MMP-9 may influence the invasiveness and recurrence of PA (*Figure 2*).^74^

### Pituitary tumour transforming gene

Human pituitary tumour transforming gene (PTTG) is an oncogene that was initially isolated from PT cells. It was identified as a securin, a protein that regulates chromosome separation, that can serve as a marker of malignancy grades in several forms of cancer, particularly endocrine malignancies such as PT.^19^

PTTG expression was evaluated in 27 recurrent ACTH-secreting PTs, in 28 non-recurrent ACTH-secreting PTs and in two normal tissues.^19^ PTTG expression was not significantly different between the recurrent group and the non-recurrent group. On the other hand, another study evaluated the expression of PTTG in 11 invasive and 11 non-invasive NFPAs and found significantly higher mRNA expression of PTTG in the invasive group compared with the non-invasive group.^22^ Similar outcomes were reported by another study, which evaluated PTTG expression in 74 PTs (48 NFPAs and 26 FPAs) and its association with recurrence over a period of 6.0 (± 3.9) years follow-up.^37^ PTTG nuclear positivity was associated with a higher risk of regrowth or recurrence of the PT (p=0.026), an association which was not statistically significant when total PTTG expression levels were analyzed. Nuclear PTTG positive expression was also positively correlated with tumour volume and suprasellar extension.^37^ Furthermore, a study evaluating PTTG expression in 38 invasive and 18 non-invasive NFPAs, found a positive rate in more than 50% of the cases, with higher indexes in invasive adenomas.^23^ The same study reported a statistically significant relationship between PTTG and invasiveness (p=0.022).^23^ No expression of PTTG was detected in the two normal pituitary tissues.^19^ In contrast with these findings, there are studies supporting PTTG as a biological marker in PAs^75^ and other studies supporting the theory that the expression of PTTG was not significantly associated with tumour invasiveness in patients with PA.^76^

*AKt = protein kinase B; Eph = ephrin receptor; FAK = focal adhesion kinase; JAK2 = Janus kinase 2; MMP = matrix metalloproteinase; PI3K = phosphatidylinositol 3-kinase; RHOA = Ras homologous A; STAT3 = signal transducer and activator of transcription 3.*

**Figure 3:**
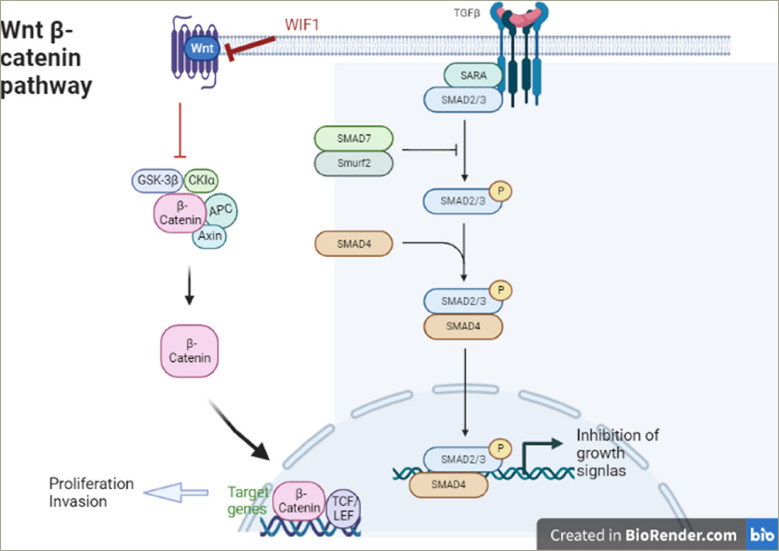
Transforming growth factor β1/Smad protein and Wnt pathway

*APC = adenomatous polyposis coli; CKIα = casein kinase Iα; GSK-3β = glycogen synthase kinase 3 β; LEF = lymphoid enhancer factor; SARA = Smad anchor for receptor activation; TCF = T cell factor; TGFβ = transforming growth factor β; WIF1 = Wnt inhibitory factor 1.*

### Fascin protein

Fascin is an actin-bundling protein that binds β-catenin and regulates cytoskeletal structures for the maintenance of cell adhesion. Fascin increases the invasiveness of cancer cells since its expression is upregulated in a spectrum of cancers.^77^ The expression of fascin protein has been assessed in a total of 30 invasive and non-invasive PTs, and higher expression was observed in invasive PTs compared with non-invasive PTs.^20^

### Immune checkpoint molecules

Immune checkpoint molecules are key receptors that inhibit the immune response and prevent its overactivation. Under normal conditions, this mechanism is responsible for maintaining tolerance to its own antigens; however, it can be used by cancer cells to avoid recognition and destruction.^78^ A number of receptors belonging to the immune checkpoint family have been discovered, including inhibitory immune checkpoints molecules such as programmed cell death ligand 1 (PD-L1), PD-L2, cytotoxic T lymphocyte-associated protein 4 (CTLA-4), CD80, CD86 and stimulatory immune checkpoint molecules such as CD27, CD40, OX40, glucocorticoid-induced tumor necrosis factor receptor-related protein (GITR) and CD137 (members of the tumour necrosis factor receptor superfamily), which exert inhibitory or stimulatory effects on immune responses. Their main role is to downregulate the immune system and promote self-tolerance by suppressing T cell inflammatory activity. Consequently, this can prevent autoimmune diseases, but it can also prevent the immune system from killing cancer cells.^77^ The importance of these molecules has led to the development of immune checkpoint blockade therapy, which has become a major weapon in anti-cancer treatment. PD-L1 is a transmembrane protein and a ligand of programmed cell death protein 1 (PD-1), and plays an important role in maintaining peripheral and central immune tolerance by interacting with PD-1. The expression of PD-L1 by tumour cells or infiltrative immune cells has been confirmed by immunohistochemical staining of various tumour tissues, such as non-small cell lung cancer, melanoma and epithelial ovarian cancer.^79^

PD-L1 expression has gained attention through many studies.^27,30,34,39,40^ PD-L1 has been investigated in 73 NFPAs (gonadotroph adenomas, silent corticotroph adenomas, NCAs), and was found to have significantly lower expression in invasive NFPAs than in non-invasive NFPAs (p<0.05), and lower expression in silent corticotroph adenoma and NCA than in gonadotroph NFPA.^34^ PD-1, PD-L1, PD-L2, CTLA-4, CD80 and CD86 were investigated in 43 recurrent or invasive PAs, 17 PTs without recurrence or non-invasive and in 12 normal pituitaries.^27^ Significantly higher levels of PD-L2 (p<0.0001), CD80 (p=0.0035) and CD86 (p=0.004) were found in recurrent or invasive PAs, but no significant difference was found in PD-1, PD-L1 and CTLA-4 between recurrent or invasive PTs and normal pituitaries.^27^ Significantly higher expression of PD-L1 (p=0.02) and PD-L2 (p<0.0001) was found in non-recurrent or non-invasive and normal pituitaries.^27^ No significant difference was found between PD-1, CTLA-4, CD80 and CD86 expression between non-recurrent or non-invasive PTs and normal pituitaries. The expression of CD86 in recurrent or invasive PTs was significantly higher compared with non-recurrent or non-invasive PTs (p=0.035); however no statistically significant differences were found between PD-1, PD-L1, PD-L2, CLTA-4 and CD80 in these groups.^27^ No significant difference of PD-1, CTLA-4, CD80 and CD86 was found between functioning PTs (n=21) and non-functioning PTs (n=11);^27^ similar findings for PD-1 were confirmed by one more study.^40^ PD-L1 was evaluated in 25 FPAs, 30 NFPAs and 10 healthy tissues and a significant increase in expression of PD-L1 was found, in PTs compared with healthy tissues (p<0.001).^30^ Interestingly, the levels of immune expression of PD-L1 in PTs ranged from negative staining to highly positive immunostaining, whereas immune expression of PD-L1 in healthy tissues were all negative staining. There was no significant difference in the expression of PD-L1 between FPAs and NFPAs. Other studies reported that PD-1 and PD-L1 showed higher expression in FPAs (p<0.01) compared with NFPAs,^35,39^ especially in growth hormone secreting adenomas.^35^

### Transforming growth factor 1, Smad proteins and Wnt inhibitory factor 1 protein

Transforming growth factor beta 1 (TGF-β1) is a cytokine that performs many cellular functions, including the control of cell growth, cell proliferation, cell differentiation and apoptosis. It is overexpressed in many human tumours, such as breast, melanoma, renal, prostatic, ovarian, haematological, cervical and brain tumours. TGF-β inhibits cell cycle progression by stopping cells from making the G1/S phase transition.^80^

Smad proteins are the main signal transducers for receptors of the TGF-β superfamily by regulating cell development and growth. Smad proteins activate TGF-β by downregulating Myc, which is a transcription factor that promotes cell growth. Myc also represses p15 and p21, which are inhibitors of Cdk4 and Cdk2, respectively.^81^ When there is no TGF-β present, a repressor complex composed of Smad3 and the transcription factors E2F4 and p107 are found in the cytoplasm. However, when the TGF-β signal is present, the complex localizes to the nucleus where it associates with Smad4, and binds to the TGF-β inhibitory element of the Myc promoter to repress its transcription.^82^ The role of TGF-β and Smad is discussed in the literature in several types of PA.^83–85^

Wnt inhibitory factor 1 (WIF1) is a lipid-binding protein encoded by the *WIF1* gene that binds to Wnt proteins and prevents them from triggering signalling (Wnt pathway off). It has been suggested that aberrant regulation of the Wnt signalling pathway is associated with tumourigenesis.^86,87^

TGF-β1 and phospho-Smad3 protein were investigated in 13 invasive somatotropinomas and 32 non-invasive somatotropinomas. TGF-β1 protein level was significantly less in the invasive than non-invasive somatotropinomas (p<0.01). Low expression of phosho-Smad3 was also correlated with invasion of somatotropinomas (p<0.01).^24^

TGF-β1 and WIF1 have been evaluated in 59 NFPAs without recurrence and in 45 NFPAs with recurrence followed for between 6 and 68 months (mean: 38.5 months). The expression of TGF-β1 and WIF1 in the recurrence group was lower than in the non-recurrence group (p<0.001). The same study also reported that NFPAs with low expression of the two proteins are more likely to recur and thus had a shorter time of recurrence (TGF-β1: 52 months; WIF1: 58 months) compared with those with high expression (TGF-β1: 65 months; WIF1: 68 months), but there was no statistically significant difference.^26^ The results of another study,^25^ which investigated TGF-β in 19 invasive NFPAs and 23 non-invasive NFPAs were in agreement with the previously mentioned studies.^24,26^ It is reported that the expression of TGF-β was significantly less in the invasive NFPAs compared with the non-invasive ones (p<0.05) (*Figure 3*).

### Glial fibrillary acidic protein

Glial fibrillary acidic protein (GFAP) is expressed by several cell types in the central nervous system.^88^ The co-expression of GFAP and cytokeratin has been investigated in 326 PAs and 13 normal anterior pituitaries.^44^ Simultaneous co-expression of GFAP and cytokeratin was demonstrated in 26 out of 326 PAs and in all 13 normal pituitaries. Furthermore, PTs with cellular co-expression of GFAP and cytokeratin were associated with a lower recurrence rate (7.7%) compared with adenomas without co-expression (17.8%).^44^

### Oestrogen receptors

The expression of oestrogen receptors (ER), ERα36 and ERα66, have been investigated in 62 prolactinomas, and it was reported that low expression of ERα36 and ERα66 was associated with tumour invasion and increased Ki-67.^45^ Moreover, low ERα66 expression was associated with dopamine-agonist resistance and enhanced tumour size.

### Galectin-3 protein

Galectin-3 is a member of the beta-galactoside-binding protein family that plays an important role in cell–cell adhesion, cell–matrix interactions, macrophage activation, angiogenesis, metastasis and apoptosis. Its expression is suggested to be a predictive biomarker of tumour aggressiveness.^89^ Galectin-3 expression has been investigated in 12 invasive prolactinomas and 24 non-invasive prolactinomas, and no significant difference was observed; a positive result for galectin-3 was observed in 45.5% of invasive tumours and 54.5% of non-invasive tumours.^28^ An association was observed between galectin-3 expression and persistence of hyperprolactinaemia.

### Cyclin A protein

Cyclin A is a member of the cyclin family that regulates progression through the cell cycle. Overexpression of cyclin A has been linked to astrocytomas’ proliferative state,^90^ reduced survival in oesophageal cancer,^91^ early relapse in prostate cancer^92^ and poorer tumour grade in oral cancers;^93^ there are limited data for NFPAs. Cyclin A expression was investigated in 15 invasive and in 16 non-invasive NFPAs, and increased expression was found in a minority of NFPAs but does not seem to be related to invasion.^29^

### S100B protein

S100B is expressed in non-endocrine cells of the pituitary, which are also described in pituitary neoplasms, suggesting they might play a role in tumourigenesis-related processes.^94–96^ The expression of S100B protein was investigated in 54 PTs and in four normal pituitaries, and it is reported that decreased expression was observed in PTs compared with normal pituitaries. Low expression of S100B was associated with a Ki-67 index ≥3, a mitosis count >2/10 per high power fields and a proliferative status, but no association was reported with invasion.^32^

### Neural cell adhesion molecule

Neural cell adhesion molecule (NCAM), also called CD56, is a homophilic-binding glycoprotein expressed on the surface of neurones, glia and skeletal muscle that has been implicated as having a role in cell–cell adhesion.^97^ NCAM expression was investigated in 16 NFPAs, eight somatotrophinomas and five normal pituitaries, and no significant difference of NCAM expression was observed either between PT and normal pituitary or NFPA and somatotrophinoma.^42^ There was also no association between NCAM expression and invasion.^42^

### Protein tyrosine phosphatase 4A3

Protein tyrosine phosphatase 4A3 (PTP4A3) is a subclass of the protein tyrosine phosphatase super family and is expressed in a range of epithelial neoplasms. Overexpression of this gene promotes cell growth. PTP4A3 expression was investigated in 34 FPAs and was expressed in more than half of the tumours (19/34); a significant association with the tumour size was observed (p=0.042).^43^

### Steroidogenic factor 1

The SF-1 protein is a transcription factor that is proposed to interact with β-catenin.^98^ Expression of SF-1 was investigated in 20 recurrent gonadotrophic PTs and 31 non-recurrent PTs, and it was reported that gonadotrophic PT with patchy SF-1 staining is more likely to recur sooner than gonadotrophic PT with diffuse staining (p=0.0007).^33^

## Discussion

Nowadays, invasiveness can be estimated radiologically and surgically, but the need to identify biomarkers that could be useful to everyday clinical management and provide a prognostic value in patients with PT is of great additive importance.

Acknowledging the lack of studies in the literature, the scarcity of the pituitary tissues and the complexity of the mechanisms of tumour growth, we have tried to outline a simple approach to understanding the importance of the various biomarkers that are used to provide prognostic information, and to assist treating clinicians with determining appropriate patient management and surveillance.

This review has shown that the expression of MCM-7, EGFR, MMP-9, PTTG, PD-1/PD-L2 and CD80/86 may be possible prognostic biomarkers for recurrent PTs, and expression of COX, ARG1, ESM1, PD-1 /PD-L2, CD80/86, MMP-9, PTTG and fascin protein may be possible prognostic biomarkers for invasive PTs. Notably, PTs with high expression of tyrosine kinase EGFR had 4.9 times higher risk of recurrence (HR 4.9). Moreover, the expression of tyrosine kinase FGFR4 was associated with the proliferative characteristic of PT and could be a marker for more aggressive tumour behaviour. Since tyrosine kinase inhibitors are a therapeutic option, these biomarkers could be of clinical value. Well-designed randomized controlled trials are needed in order to understand their impact on the pathophysiology of PT and, consequently, on recurrence or invasion, before using them in everyday clinical management. From this perspective, the most important biomarkers appear to be MMP-9, PD-1/ PD-L2 and CD80/86.

Low expression of other biomarkers, namely TGF-β1, WIF1, and co-expression of the GFAP and cytokeratin, was associated with recurrence of PT; also, low expression of TGF-β1, phospho-Smad, ERα36 and ERα66 was associated with invasion of PT. Higher expression of these biomarkers and TIMP-1 was noted in non-recurrent or non-invasive PTs. With caution, we could hypothesize that the downregulation of the abovementioned biomarkers may be associated with tumour growth.

Expression of additional biomarkers (i.e. EPH, PD-1/PD-L1, CTLA-4, galectin 3, cyclin A, S100 protein, NCA, PTP4A3 and SF-1) did not show a clear association with recurrence or invasion of PT. However, before we reject them as unimportant, additional studies might clarify their role in proliferation, invasion or recurrence, particularly for the druggable molecules such as EPH and PD-1/PD-L1.^27,30,41^

**Figure 4:**
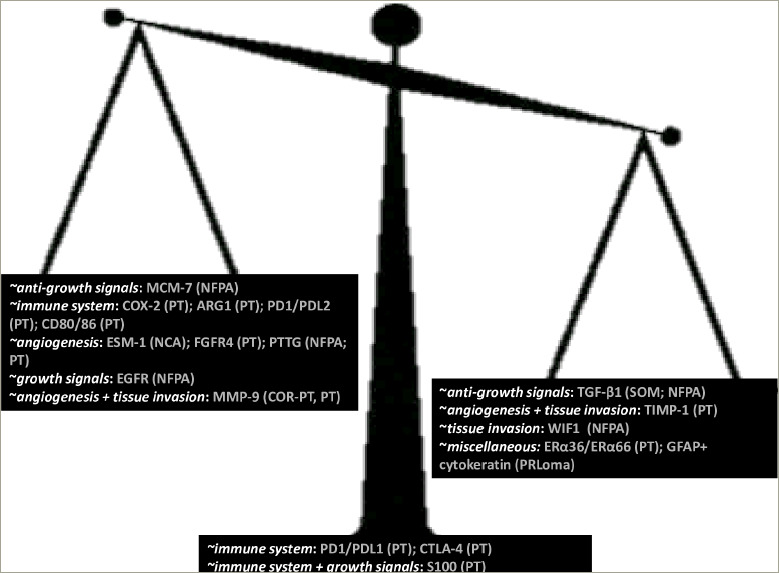
Positive and negative prognostic biomarkers in pituitary tumour

*In the left scale, the biomarkers with positive association with disease recurrence and/or invasion are shown. In the right scale, the biomarkers with negative association with recurrence and/or invasion are shown. In the base of the scale, the biomarkers with no association with disease recurrence and/or invasion are shown. The evaluated biomarkers were sorted according to the evaluation of a minimum of 50 pituitary species for each marker in one or more studies evaluated in the present review.*

*ARG1 = arginase 1; COR-PT = corticotroph pituitary tumors; COX-2 = cyclooxygenase 2; CTLA-4 = cytotoxic T-lymphocyte-associated protein 4; EGFR = epidermal growth factor receptor; ER = oestrogen receptor; ESM1 = endothelial cell-specific molecule 1; FGFR4 = fibroblast growth factor receptor 4; GFAP = glial fibrillary acidic protein; MCM-7 = minichromosome maintenance protien 7; MMP-9 = matrix metalloproteinase-9; NCPA = null-cell pituitary adenoma; NFPA = non-functioning pituitary adenoma; PD1 = programmed cell death protein 1; PDL = programmed cell death ligand; PT = pituitary tumour; PTTG = pituitary tumour transforming gene; PRLoma = prolactinoma; SOM = somatotroph adenoma; TGF-β1 = transforming growth factor β1; TIMP-1 = tissue inhibitor of metalloproteinase 1; WIF1 = Wnt inhibitory factor 1.*

In aggregate, this review attempts to summarize novel biomarkers, that would predict PT behaviour (i.e. invasiveness, aggressiveness, metastases). Many studies with sufficient number of species, adequate follow-up period and consistent outcomes have drawn attention to MMP-9, MCM-7 and FGFR (tyrosine kinase pathway). Moreover, expression of PTTG (hyperactivation of mammalian target of the rapamycin [mTOR] signalling) has drawn the attention of many researchers. Although studies do not show consistent results, the majority of them have reported an association with either recurrence or tumour growth or suprasellar extension or invasiveness.

*Figure 4* depicts the biomarkers that have been investigated in more than 50 pituitary specimens. Some biomarkers were found to be positively correlated with disease recurrence and invasion in PTs, for example, COX-2, ARG1, PD-1/PD-L2, TGF-β1 and CD80/86, all of which have a role in evasion of the immune system; FGFR and PTTG have a role in sustained angiogenesis; and MMP-9 has a role in sustained angiogenesis and tissue invasion.

In NFPA, the biomarkers that were found to be positively correlated with disease recurrence and invasion were: MCM-7 protein that has a role in the insensitivity to anti-growth signals, EGFR that modulates self-sufficiency in growth signals, and PTTG that has a role in sustained angiogenesis. In null-cell PT, ESM1, which has a role in sustained angiogenesis, was positively correlated with invasion. In corticotroph PT, MMP-9 was more highly expressed in recurrent versus non-recurrent PT. On the other hand, a number of other biomarkers may be negatively correlated with disease recurrence and invasion in PT, such as TIMP-1, which has a role in sustained angiogenesis and tissue invasion, and ERα36 and ERα66. In NFPA, TGF-β1, an inhibitor of insensitivity to anti-growth signals, and WIF1, which has role in tissue invasion, were negatively correlated with disease recurrence and invasion. In prolactinomas, the co-expression of GFAP and cytokeratin was negatively correlated with disease recurrence of PT. Finally, no association with recurrence and invasion of PT was found for S100, as opposed to the expression of PD-1/PD-L1 that had no consistent association with recurrence and invasion or with the functionality of PT.

This review highlights the necessity to expand our current knowledge on PT pathogenesis by utilizing molecular and pathological tools. The progress of molecular biology by the use of next-generation sequencing includes genomics, methylomics, transcriptomics, proteomics and glycomics, which can be integrated into the term 'multiomics'. This progress will lead to a new era of therapeutics by firstly identifying the target (i.e. biomarker) of a tumour, and then targeting the treatment to the identified biomarkers for individualized treatment regimens.^99,100^ This has already been seen in somatotroph PTs, where the different immunohistochemical profiles may assist with the identification of subgroups of patients that may benefit from similar treatments .^100^ In addition, it could identify genetic alterations that may have an impact on the outcome of different therapies by targeting specific molecular pathways. An important step in this direction was introduced with the new classification of PTs that included transcription factors.^101^ A recent seminal study evaluated 134 patients with functioning PTs via multiomics, such as chromosomal alterations, miRNomics (MicroRNA Biology and Computational Analysis), methylomics and RNA transcriptomics.^98^ According to methylomics, these tumours were classified into three groups, combining the collapsed CpGs and the secretion of the tumours: met1 correlated with somatotrophs, lactotrophs and thyrotrophs; met2 correlated with gonadotrophs; and met3 correlated with corticotrophs. According to miRNomics, these tumours were classified into four groups based on microRNA (miRNA) clusters as prolactin-secreting (miR-1), growth hormone (miR-2), ACTH (miR-3) and follicle-stimulating hormone/ luteinising hormone tumours (miR-4). Finally, according to transcriptomics (pangenomic analysis: somatic mutations, chromosomal alterations, miRNome, methylome, transcriptome), these tumours were classified into six clusters: ubiquitin-specific protease 8 wild-type corticotrophs, overt Cushing corticotrophs (t1 cluster) that appeared more aggressive; lactotroph (t2) with higher dopamine receptor 2 expression, silent corticotroph with a gonadotroph signature (t3); gonadotroph and null-cell (t4); sparsely granulated somatotroph with thyrotroph and plurihormonal PIT1-positive (t5); thyrotrophs, somatotrophs and mixed growth hormone-prolactin (t6).^98^

## Conclusion

In this review we have summarized important biomarkers that could provide prognostic information, thus assisting clinicians in a more efficient management and surveillance of these neoplasms. PTs are mostly a benign disease with a long survival. However, treatment complications may alter the quality of life of the patients harbouring these neoplasms, creating an increased need for effective and safe management based on specific molecular tools. A limited number of molecular targets have been studied in the context of PT recurrence and invasion. Further investigation of the most relevant of these biomarkers by well-designed interventional studies will result in a better understanding of the molecular profile of PT. This could meet the increased need of treatable molecular targets, and lead to a more personalized approach to the treatment of PTs.
